# Successful treatment with risperidone increases 5‐HT 3A receptor gene expression in patients with paranoid schizophrenia – data from a prospective study

**DOI:** 10.1002/brb3.798

**Published:** 2017-08-13

**Authors:** Hongying Chen, Yong Fan, Lei Zhao, Yong Hao, Xiajun Zhou, Yangtai Guan, Zezhi Li

**Affiliations:** ^1^ Department of Neurology Ren Ji Hospital School of Medicine Shanghai Jiao Tong University Shanghai China; ^2^ Department of Psychiatry Changning Mental Health Center Shanghai China; ^3^ Department of Psychiatry Qingdao Mental Health Center Qingdao Shandong China

**Keywords:** 5‐HT 3A receptor, paranoid schizophrenia, risperidone, treatment

## Abstract

**Introduction:**

The relationship between peripheral 5‐HT3A receptor mRNA level and risperidone efficiency in paranoid schizophrenia patients is still unknown.

**Methods:**

A total 52 first‐episode and drug‐naive paranoid schizophrenia patients who were treated with risperidone and 53 matched healthy controls were enrolled. Patients were naturalistically followed up for 8 weeks. Positive and Negative Syndrome Scale (PANSS) was applied to assess symptom severity of the patients at baseline and at the end of 8th week.

**Results:**

There was no difference in 5‐HT3A receptor mRNA level between paranoid schizophrenia patients and healthy controls at baseline (*p *= .24). Among 47 patients who completed 8‐week naturalistic follow‐up, 37 were responders to risperidone treatment. 5‐HT3A receptor mRNA level of paranoid schizophrenia patients did not change in overall patients after 8‐week treatment with risperidone (*p *= .29). However, 5‐HT3A receptor mRNA level in responders increased significantly (*p *= .04), but not in nonresponders (*p *= .81).

**Conclusions:**

Successful treatment with risperidone increases 5‐HT3A receptor gene expression in patients with paranoid schizophrenia, indicating that 5‐HT3A receptor may be involved in the mechanism of risperidone effect.

## 1 INTRODUCTION

Schizophrenia is a chronic and disabling mental disorder affecting 1–2% of the population worldwide, characterized by heterogeneous clinical features (Lu et al., 2012; Wey, Loh, Doss, Abu Bakar, & Kisely, 2016). Although the underlying etiology of schizophrenia is still poorly understood, lines of evidence suggest that dysfunction in 5‐hydroxytryptamine (serotonin) processes involves in the pathophysiology of schizophrenia (Ellenbroek & Prinssen, 2015; Wooley & Shaw, 1954). Furthermore, another evidence is that the serotonin system plays a critical role in the efficiency of second generation antipsychotics, though binding much strongly with the serotonin system than dopamine system (Lieberman et al., 1998).

Within the serotonin system, only 5‐HT3 receptor is a ligand‐gated ion channel, whereas the others are G‐protein‐coupled receptors. Among five identified 5‐HT3 receptor subtypes (A‐E), only subtypes A, B, and C are expressed in the brain (Niesler, Frank, Kapeller, & Rappold, 2003). While 5‐HT3A is the most important subtype of the receptor, 5‐HT3B subtype is functional only coexpressed with 5‐HT3A subtype (Davies et al., 1999) and the 5‐HT3C subtype may combine with 5‐HT3A to work (Niesler et al., 2007).

Mounting evidence has demonstrated that 5‐HT3A receptor was involved in the pathophysiology of schizophrenia. First, the 5‐HT3A receptor encoding gene is located at chromosome 11q23.1, which is one of the most important regions showing strong evidence for linkage to schizophrenia (Levinson et al., 1998; Maziade et al., 1995). Second, 5‐HT3A is involved in the release regulation of dopamine and other neurotransmitters including acetylcholine and gamma‐aminobutyric acid (GABA), which are thought to be the target for some antipsychotics. Third, some previous studies revealed that 5‐HT3A receptor could affect the efficiency of the second generation antipsychotics (Rajkumar et al., 2012; Souza, de Luca, Meltzer, Lieberman, & Kennedy, 2010). Recently, Gu et al. (2008) reported that the polymorphism of 5‐HT3A receptor may be a useful predictor of therapeutic response to risperidone, which is one of the most commonly used atypical antipsychotics in China. However, the positive relationship between the polymorphism of 5‐HT3A receptor and risperidone efficiency was not observed in another research (Schuhmacher et al., 2009). The most possible reason for these inconsistent results may be the heterogeneous clinical features of schizophrenia, for the different subtypes of schizophrenia may have different genetic basis (Lin et al., 2013). On the other hand, the most important thing is that the 5HT3A receptor polymorphism could not be associated with associated with the representation on mRNA or plasma levels in humans. Together, few studies examine the 5‐HT3A receptor level in schizophrenia patients, thus far, and its significance in these patients is not clear. The aim of this study was to examine peripheral 5‐HT3A receptor mRNA levels in paranoid schizophrenia patients; and determine the relationship between peripheral 5‐HT3A receptor mRNA level and risperidone efficiency.

## 2 PATIENTS AND METHODS

### 2.1 Patients

Inpatients and outpatients were enrolled in Changning Mental Health Center during the period from January 2008 to January 2010. The first‐episode and drug‐naïve Chinese Han population patients aging from 18 to 60 years old, meeting the criteria of the Diagnostic and Statistical Manual of Mental Disorders, Fourth Edition (DSM‐IV) for paranoid schizophrenia were enrolled in this study. Patients who had a lifetime diagnosis of schizoaffective disorder, bipolar disorder, or other psychotic disorders were excluded.

All patients in this study were interviewed by two experienced psychiatrists using Mini International Neuropsychiatric Interview (MINI). MINI is a brief structured interview for Axis I diagnosis of major psychiatric disorders in International Classification of Diseases‐Tenth Edition (ICD‐10) and Diagnostic and Statistical Manual of Mental Disorders‐Fourth Edition (DSM‐IV). The severity of the patients were assessed using Positive and Negative Syndrome Scale (PANSS) (interrater reliability, Kappa = 0.87).

Age‐ and gender‐matched healthy controls were recruited from university and community. Individuals with any major Axis I disorders and family history of mental disorder (had a lifetime diagnosis of schizoaffective disorder, bipolar disorder, or other psychotic disorders) were excluded. Eventually, total of 52 patients with a mean age of 31.2 ± 4.2 years (age from 24 to 47) (39 males and 13 females) and matched 53 healthy controls with a mean age of 31.4 ± 4.1 years (age from 24 to 48) (35 males and 18 females) were recruited in this study. There were no significant differences in age and gender between patients and healthy controls (*t *= 0.27, *df* = 103, *p *= .79; χ^2^ = 1.01, *df* = 1, *p *= .31, respectively).

All procedures were reviewed and approved by Institutional Review Boards of Changning Mental Health Center. This study was conducted in accordance with the Helsinki Declaration as revised 1989 and written informed consent was obtained from each participant before any study‐related procedures were carried out.

### 2.2 Naturalistic follow‐up

This study was naturalistic observation study. Patients only treated with risperidone were recruited after written informed consent was obtained. Patients started from an initial dose of 1 mg daily and gradually increasing to 4–6 mg daily in 6–7 days. Benzhexol hydrochloride could be used for extrapyramideal side effects. Benzodiazepines could be used shortly for insomnia. Patients were followed up for 8 weeks, and the reduction rate of PANSS scores was assessed at the end of the 8th week. The reduction rate of PANSS scores  =  (scores before treatment ‐ scores after treatment)/(scores before treatment − 30) × 100%. Patients with schizophrenia were divided into responders and nonresponders according to the reduction rate of PANSS. Responders were the patients with the reduction rate of PANSS higher or equal to 25%, whereas nonresponders were those with reduction rate of PANSS score less than 25% (Leucht, Davis, Engel, Kissling, & Kane, 2009).

### 2.3 Peripheral blood collection and cDNA preparation

Peripheral venous blood was collected from the fasting healthy controls and patients (at baseline/after 8‐week treatment with risperidone) in the morning. cDNA was prepared as previous described (Li et al., 2014). In brief, total RNA was extracted from blood samples using the QIAamp RNA blood Mini Kit (Qiagen, Chatsworth, CA, USA), and was afterward treated with DNase (Qiagen). The complementary DNA (cDNA) was synthesized from total RNA by Omniscript Reverse Transcription Reagents (Qiagen) and a random primer according to the manufacturer's protocols.

### 2.4 Detection for 5‐HT3A receptor mRNA level

Quantitative RT‐PCR was performed by TaqMan gene expression assay using ABI Prism 7900 sequence detection system with a 384‐well format, according to the manufacturer's protocol. 5‐HT3A receptor mRNA level was determined as previous described (Li et al., 2014). In brief, human Glyceraldehyde‐3‐phosphate dehydrogenase (GAPDH, Applied Biosystems 1997, CA, USA) was used as an endogenous control to normalize the mRNA expression level of target genes. All specific primers, TaqMan probes/primers for target gene and GAPDH were obtained from Applied Biosystems. Quantitative RT‐PCR reaction was as follows: 50° C for 2 min and 95° C for 10 min, then 95° C for 50 cycles of 10s, 59° C for 1 min. Each subject was repeated in triplicate. The experiment performers were blind to all the clinic data.

### 2.5 Data analysis and statistical tests

Data were collected and analyzed with Sequence Detector Software version 2.1 (Applied Biosystems). As for real‐time PCR, the gene expression levels were represented as Ct value, which was defined as the threshold cycle of PCR when significant increase is first detected in the fluorescence signal [16]. The Comparative Ct Values (ΔCt) were applied for relative expression of target gene, and 2^‐ΔCt^ represented the relative expression levels. The ΔCt values were obtained by subtracting the average Ct value of GAPDH of each sample from the average Ct value of target gene of each sample (Li et al., 2014).

Statistical Package for Social Sciences (SPSS, version 17.0; Chicago, Ill) was used to analyze the data. Normal distribution of the data was analyzed though One‐sample Kolmogorov–Smirnov Test. Demographic data including gender and age were analyzed by using chi‐square and t‐test, respectively. Independent t‐test applied to analyze the difference of 5‐HT3A receptor mRNA level (2^−ΔCt^) between patients and healthy controls. Paired t‐test was applied to analyze whether 5‐HT3A receptor mRNA levels change after treatment with risperidone. Linear regression models were performed to analyze the association between 5‐HT3A receptor mRNA level and PANSS scores on admission, including confounding factors including age and gender to balance their effects. Also, the association between the change in 5‐HT3A receptor mRNA level and total PANSS scores was analyzed using the same method. *p* value <.05 was considered statistically significant.

## 3 RESULTS

Total 52 first‐episode drug‐naive paranoid schizophrenia patients with total PANSS score of 66.9 ± 9.4 were enrolled. During 8‐week naturalistic follow‐up, one patient withdrew due to extrapyramideal side effects; one patient withdrew due to QT prolongation; three patients withdrew due to early discharge from hospital. Finally, a total of 47 patients completed 8‐week naturalistic follow‐up. After treatment with risperidone for 8 weeks, the PANSS score of the patients was 49.6 ± 8.4. Among 47 patients, 37 patients with the reduction rate of PANSS scores equal or higher than 25% were responders, whereas the rest of the patients with the reduction rate of PANSS scores lower than 25% were nonresponders. The demographic data between responder and nonresponder group were comparable, including age (31.4 ± 4.8 vs. 31.0 ± 2.8, *df* = 45, *p *= .81), gender (χ^2^ = 2.12, *df* = 1, *p *= .35), and total PANSS score (66.2 ± 9.1 vs. 67.4 ± 10.7, *df* = 45, *p *= .73).

### 3.1 The effect of risperidone on 5‐HT3A receptor mRNA level

The 5‐HT3A receptor mRNA level of paranoid schizophrenia patients at the baseline was 0.23 ± 0.04. In contrast, 5‐HT3A receptor mRNA level of healthy controls was 0.24 ± 0.05. There was no difference in 5‐HT3A receptor mRNA level between paranoid schizophrenia patients and healthy controls (*t *= 1.19, *df* = 103, *p *= .24).

When the patients were divided into responders (*n *= 37) and nonresponders (*n *= 10), the baseline 5‐HT3A receptor mRNA level was not different between these two subgroups. The 5‐HT3A receptor mRNA level in responders was 0.23 ± 0.04 and in nonresponders was 0.24 ± 0.03 (*t *= 1.07, *df* = 45, *p *= .29).

**Figure 1:**
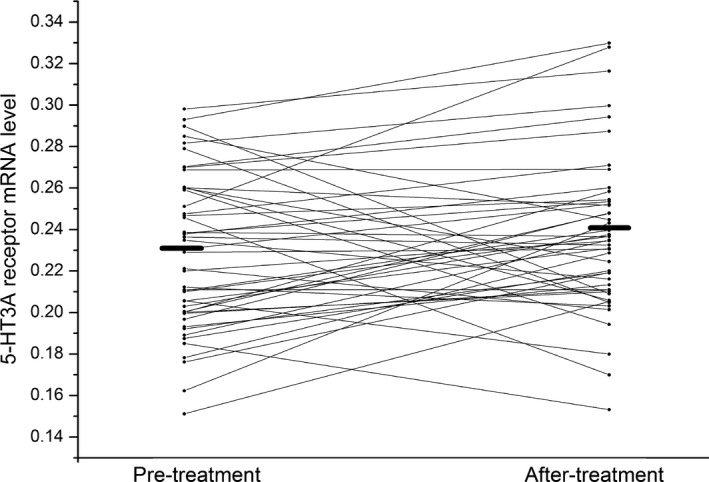
The effect of risperidone on 5‐HT3A receptor mRNA level. The left black line represents average 5‐HT3A receptor mRNA level at the baseline, and the right black line represents average 5‐HT3A receptor mRNA level at the end of 8 week after risperidone treatment

**Figure 2:**
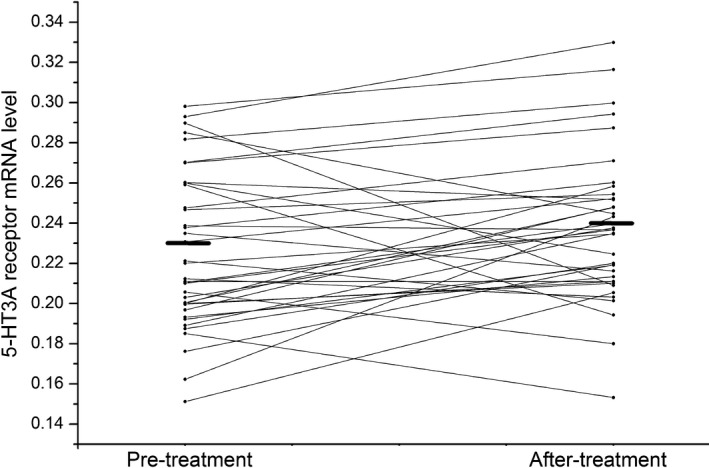
The effect of risperidone on 5‐HT3A receptor mRNA level in responders. The left black line and the right black line represent average 5‐HT3A receptor mRNA level at the baseline and the end of 8 week, respectively, in responder group

**Figure 3:**
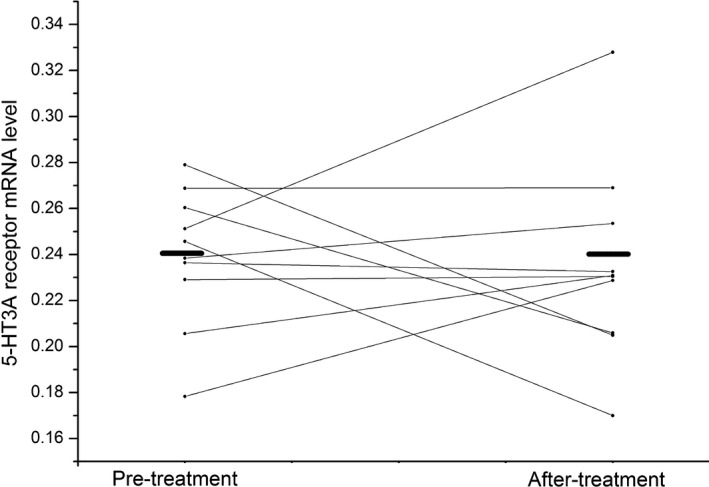
The effect of risperidone on 5‐HT3A receptor mRNA level in nonresponders. The left black line and the right black line represent average 5‐HT3A receptor mRNA level at the baseline and the end of 8 week, respectively, in nonresponder group

After treatment with risperidone for 8 weeks, 5‐HT3A receptor mRNA level did not change in overall patients (pretreatment 0.23 ± 0.04 vs. after treatment 0.24 ± 0.04) (*t *= 1.52, *df* = 46, *p *= .14) (shown in Figure 1). However, 5‐HT3A receptor mRNA level increased marginally significantly in responders (pre‐treatment 0.23 ± 0.04 vs. after treatment 0.24 ± 0.04) (*t *= 2.10, *df* = 36, *p *= .04) (shown in Figure 2). In contrast, in nonresponders, there was no difference in 5‐HT3A receptor mRNA level at the end of 8th week compared to baseline levels (pretreatment 0.24 ± 0.03 vs. after treatment 0.24 ± 0.04) (*t *= 0.24, *df* = 9, *p *= .81) (shown in Figure 3).

### 3.2 Association between 5‐HT3A receptor mRNA level and PANSS scores

Linear regression models were used to test the association between baseline 5‐HT3A receptor mRNA level and PANSS scores. These models were composed of independent (including age, gender, and PANSS score) and dependent variables (5‐HT3A receptor mRNA level). The baseline 5‐HT3A receptor mRNA level did not correlate with total PANSS scores (*F *= 1.86,*p *= .15), positive scale scores (*F *= 1.44,*p *= .24), negative scale scores (*F *= 1.48,*p *= .23) or general psychopathology scale scores (*F *= 1.83,*p *= .15) in patients on admission. (Table 1).

**Table 1:** Association between the 5‐HT3A receptor mRNA level and PANSS scores

Independent or covariate variable	Total^a^		Positive^b^		Negative c		General d	
	B	*p*	B	*p*	B	*p*	B	*p*
The reduction rate of PANSS	−0.001	.26	0.000	.67	−0.001	.60	−0.001	.27
Age	0.002	.05	0.002	.06	0.002	.07	0.002	.05
Gender	0.000	.98	0.002	.87	0.005	.67	0.001	.27

a Association between the 5‐HT3A receptor mRNA level and the reduction rate of total PANSS scores.

b Association between the 5‐HT3A receptor mRNA level and the reduction rate of positive scale scores.

c Association between the 5‐HT3A receptor mRNA level and the reduction rate of negative scale scores.

d Association between the 5‐HT3A receptor mRNA level and the reduction rate of general psychopathology scale scores.

### 3.3 Association between the changes in 5‐HT3A receptor mRNA level and reduction rate of total PANSS scores

After risperidone treatment for 8 weeks, the change in 5‐HT3A receptor mRNA level was not associated with the reduction rate of total PANSS scores in patients (*F *= 0.80, *p *= .46) (shown in Table 2). In subgroups, the change in 5‐HT3A receptor mRNA level was not associated with the reduction rate of total PANSS score neither in the responders (*F *= 0.57, p =* *.64), nor in nonresponders (*F *= 0.47, p =* *.71) (shown in Table 2).

**Table 2:** Association between the changes of 5‐HT3A receptor mRNA level and reduction rate of total PANSS scores

Covariate variable	SZ Group (*N *= 47)		Responders (*N *= 37)		Nonresponders (*N *= 10)	
	B	*p*	B	*p*	B	*p*
The reduction rate of PANSS	0.04	.25	0.03	.57	0.16	.75

## 4 DISCUSSION

Although the second generation antipsychotics have a considerable effect o*n *symptoms of schizophrenia, to date, the mechanism underlying this effect is not sufficiently clear. Risperidone, the most commonly used second generation antipsychotic in China, was reported that its effect was associated with 5‐HT3A receptor. Previous studies examined the association between risperidone efficiency and the polymorphism of 5‐HT3A receptor. Gu et al. (2008) found 5‐HT3A receptor polymorphism may be a potential predictor of risperidone response; whereas Schuhmacher et al. (2009) found no correlation between them. Recently, Jajodia et al. (2015) conducted a meta‐analysis comparing their data with data from the Schizophrenia Psychiatric Genome‐Wide Association Study Consortium (PGC‐SCZ) and combined analysis of sporadic case–control association and a transmission disequilibrium test in familial samples from South Indian population. Finally they identified three associations including a functional promoter variant of HTR3A (rs1062613).

However, few studies examined the 5‐HT3A receptor mRNA level in schizophrenia patients and its association with risperidone effectiveness. In this study, we compared the 5‐HT3A receptor mRNA level between paranoid schizophrenia patients and matched healthy controls. Then we observed the patients treated with risperidone to examine the correlation between the 5‐HT3A receptor mRNA level and risperidone response.

We found that there was no difference in 5‐HT3A receptor mRNA level between paranoid schizophrenia patients and matched healthy controls, and risperidone did not change the 5‐HT3A receptor mRNA level in paranoid schizophrenia patients. Interestingly, however, when these patients were divided into responders and nonresponders, 5‐HT3A receptor mRNA level increased marginally significantly in responders, but not in nonresponders. It indicated that treatment resistance to risperidone might contribute to unaltered 5‐HT3A receptor levels, while increasing 5‐HT3A receptor mRNA level associated with successful treatment with risperidone. Results also suggested that 5‐HT3A receptor was involved in risperidone effectiveness in paranoid schizophrenia patients. The mechanism of the relationship between 5‐HT3A receptor and risperidone response remain unclear. Previous studies demonstrated the activation of 5‐HT3A receptor could increase dopamine release (Liu, Thielen, Rodd, & Mcbride, 2006), and the elevation of dopamine might contribute to the improvement of schizophrenic symptoms (Díaz‐Mataix et al., 2005). Furthermore, lines of evidence indicated the ability of 5‐HT3 receptors to modulate the release of acetylcholine and GABA (gama‐amino butyric acid) (Choi et al., 2007), which were thought to be involved in antipsychotics efficiency (Meltzer et al., 2013). Whether risperidone functions though upregulating 5‐HT3A receptor, thus inducing the release of the neurotransmission is worthy of further investigation.

Up to date, few studies examined the 5‐HT3A receptor level in schizophrenia patients, and its association with antipsychotics efficiency. Shariati et al. demonstrated that atypical antipsychotic olanzapine decreased 5‐HT3A receptor level, but haloperidol had no effect on 5‐HT3A receptor level (Shariati et al., 2009). However, they did not examine the difference in 5‐HT3A receptor level between schizophrenia patients and healthy controls; and they did not divide the patients into responders and nonresponders to further examine different risperidone effects on 5‐HT3A receptor level between those two groups. In addition, the different subtype of schizophrenia patients might have different genetic background. Thus, drug‐naive first‐episode paranoid schizophrenia patients were recruited in this study to improve the homogeneity of the samples.

The most important limitation of our study is that we could not examine 5‐HT3A receptor level in central nervous system. Up to date, no postmortem study was carried out on 5‐HT3A receptor level in schizophrenia patient. However, previous studies demonstrated that serotonin receptors can be expressed and can function in brain as well as in periphery (Grimaldi & Fillion, 2000). Furthermore, other studies indicated the correlation between brain and peripheral blood in serotonin receptors expression (Sullivan, Fan, & Perou, 2006). Second, in this study, total 47 patients completed 8‐week naturalistic follow‐up. However, only three patients had the reduction rate of PANSS scores equal or higher than 50%. Thus, we set the threshold of responder as the reduction rate of PANSS higher or equal to 25% according to previous study and recommendations (Leucht et al., 2009). The reason for lower proportion of patients with reduction rate of PANSS scores equal or higher than 50% in this study may be the small sample size, and further multiple‐size study and larger sample size need to be recruited to validate our result. It is well‐known that clinical response to antipsychotic treatment in schizophrenia is a complex phenomenon, involving biological and social factors, that cannot be ruled out in this observational study. Thus, further study should be performed including multiple confounding factors in larger samples. Third, this study only studies the association between 5‐HT 3A receptor gene expression and risperidone effect. Many other molecules besides 5‐HT3A can affect a patient's responsiveness to antipsychotics such as DRD2 (‐141C Ins/Del) and its neighboring ANKK1 (Taq1A), plus metabolic enzymes such as CYP2D6 (Almoguera et al., 2013; Miura et al., 2016; Wang et al., 2016). Further study should be performed to examine the effects of interaction of these genes on 5‐HT 3A receptor gene expression and risperidone effect.

In summary, our study suggested a putative association between the changes in 5‐HT3A receptor mRNA level and the clinical response to risperidone in paranoid schizophrenic patients. Successful treatment with risperidone increases 5‐HT 3A receptor gene expression in patients with paranoid schizophrenia indicating that 5‐HT3A receptor may be involved in the mechanism of risperidone effect on paranoid schizophrenia patients.

## CONFLICT OF INTERESTS

The authors do not have any financial or nonfinancial correlation which might pose a conflict of interest with this paper.

## AUTHORS’ CONTRIBUTIONS

Li Zezhi and Guan Yangtai: Designed the study; Chen Hongying: Wrote the draft manuscript; Hao Yong and Zhang Yi: Did the experiments. All authors have contributed to approve the final manuscript.

## ROLE OF THE SPONSOR

The funder had no role in the design, collection, management, analysis, and approval of manuscript or decision to submit the manuscript for publication.
